# Understanding resilience of female adolescents towards teenage pregnancy: a cross-sectional survey in Dar es Salaam, Tanzania

**DOI:** 10.1186/s12978-017-0338-x

**Published:** 2017-06-26

**Authors:** Constanze Pfeiffer, Collins K Ahorlu, Sandra Alba, Brigit Obrist

**Affiliations:** 1 Department of Epidemiology & Public Health (EPH), Swiss Tropical and Public Health Institute (Swiss TPH), Socinstr. 57, P.O. Box 4002, Basel, Switzerland; 2 University of Basel, Petersplatz 1, 4003 Basel, Switzerland; 3 Noguchi Memorial Institute for Medical Research, College of Health Sciences, University of Ghana, P.O Box LG581, Legon, Ghana; 4 Institute of Social Anthropology, University of Basel, Münsterplatz 19, 4051 Basel, Switzerland; 5 KIT Biomedical Research, Royal Tropical Institute (KIT), Meibergdreef 39, Amsterdam, 1105 AZ The Netherlands

**Keywords:** Resilience, Adolescents, Sexual and reproductive health, Quantitative methods, Tanzania, Urban health

## Abstract

**Background:**

In Tanzania, teenage pregnancy rates are still high despite the efforts being made to reduce them. Not enough is known about how adolescents experience and cope with sexuality and teenage pregnancy. Over the past few decades, most studies have focused on vulnerability and risk among youth. The concept of ‘*reproductive resilience*’ is a new way of looking at teenage pregnancy. It shifts the perspective from a deficit-based to a strength-based approach. The study presented here aimed to identify factors that could contribute to strengthening the reproductive resilience of girls in Dar es Salaam, Tanzania.

**Methods:**

Using a cross-sectional cluster sampling approach, 750 female adolescents aged 15–19 years were interviewed about how they mobilize resources to avoid or deal with teenage pregnancy. The main focus of the study was to examine how social capital (relations with significant others), economic capital (command over economic resources), cultural capital (personal dispositions and habits), and symbolic capital (recognition and prestige) contribute to the development of adolescent competencies for avoiding or dealing with teenage pregnancy and childbirth.

**Results:**

A cumulative competence scale was developed to assess reproductive resilience. The cumulative score was computed based on 10 competence indicators that refer to the re- and pro-active mobilization of resources. About half of the women who had never been pregnant fell into the category, ‘high competence’ (50.9%), meaning they could get the information and support needed to avoid pregnancies. Among pregnant women and young mothers, most were categorized as ‘high competence’ (70.5%) and stated that they know how to avoid or deal with health problems that might affect them or their babies, and could get the information and support required to do so. Cultural capital, in particular, contributed to the competence of never-pregnant girls [OR = 1.80, 95% CI = 1.06 to 3.07, *p* = 0.029], pregnant adolescents and young mothers [OR = 3.33, 95% CI = 1.15 to 9.60, *p* = 0.026].

**Conclusions:**

The reproductive resilience framework provides new insights into the reproductive health realities of adolescent girls from a strength-based perspective. While acknowledging that teenage pregnancy has serious negative implications for many female adolescents, the findings presented here highlight the importance of considering girls’ capacities to prevent or deal with teenage pregnancy.

## Plain English summary

Despite efforts to reduce teenage pregnancy rates in Tanzania, they are still high. Not enough is known about how female adolescents experience and cope with sexuality and teenage pregnancy. Over the past few decades, most studies have focused on vulnerability and risk among youth. The concept of ‘*reproductive resilience*’ is a new way of looking at teenage pregnancy. It shifts the perspective from a deficit-based to a strength-based approach. The study presented here aimed to identify factors that could contribute to strengthening the reproductive resilience of girls in Dar es Salaam, Tanzania.

Using a cross-sectional cluster sampling approach, 750 female adolescents aged 15–19 years were interviewed about how they mobilize resources (such as social, economic, cultural, and symbolic capital) to avoid or deal with teenage pregnancy.

About half of the women who had never been pregnant fell into the category, ‘high competence’ (50.9%), meaning they could get the information and support needed to avoid pregnancies. Among pregnant women and young mothers, most were categorized as ‘high competence’ (70.5%), and stated that they know how to avoid or deal with health problems that might affect them or their babies and could get the required information and support. Cultural capital, in particular, contributed to the competence of never-pregnant girls, pregnant adolescents and young mothers.

The reproductive resilience framework provides new insights into the reproductive health realities of adolescent girls. The findings highlight the importance of considering girls’ capacities to deal with teenage pregnancy.

## Background

Young people hold the key to the future, yet they face multiple and complex challenges. From a public health perspective, a key concern is the sexual and reproductive health of youth [1]. According to the United Nations (UN) the term “young people” is used for the age range 10 to 24. This group can be divided into three subgroups: younger adolescents (10 to 14 years); older adolescents (15 to 19 years); and youth (15 to 24 years) [1]. Adolescents especially in low-and middle-income countries are considered to be one of the most at-risk groups as far as sexual and reproductive health risks are concerned [1–4]. Pregnancy exposes adolescent women to medical, social and economic threats, as they have a high risk of dying in childbirth, of being socially excluded and of living in poverty as single mothers [2].

Due to rapid urbanization in developing countries, many adolescents grow up in urban areas. Despite the high and growing number of urban youth, not enough is known about their health and their sexual and reproductive health, in particular [5, 6].

In the past, adolescents have often been represented as being very vulnerable to sexual and reproductive health risks [1–3, 7–9]. Such thinking is rooted in a developmentalist framework that constructs adolescence as a separate stage of development during which adolescents are no longer children, but not yet adults [10]. This Western notion of adolescence [11], however, ignores the fact that childhood and adolescence mean different things in different contexts [10]. In Tanzania, for instance, marital status and motherhood shape whether adolescents are regarded as grown-ups or not.

Nevertheless, the developmentalist discourse is still reflected in some public health discussions and influences policy and practice [10]. Recently, however, discussions around the post-2015 health agenda for women, children, and adolescents highlighted the importance of a paradigm shift from problem-oriented approaches towards those that emphasize resilience and capacity among young people [12].

While this study does not ignore the various risks related to teenage pregnancy, it argues that by focusing only on adolescents’ problems or weaknesses, their strengths and capacities might be overlooked [13, 14]. In line with Harpham, it contends that, ‘*we need to know what to strengthen among low-income urban populations to protect and promote their health, and how to strengthen it. This requires information about resilience rather than vulnerability*’ ([5]: p. 115).

A strength-based approach focusing on resilience has a long history in child development psychology [15–21]. Our current understanding of resilience comes from a small community of Western-trained psychologists and social workers in the United States and Europe [22]. Only in the last few years have psychologists expanded their research to include low- and middle-income countries [22–24]. Recently, a group of social scientists from Switzerland conceptualized resilience from a social science perspective [25] and proposed a multi-layered social resilience framework. Drawing on theories of structuration and social constructivism, they acknowledge that resilience is a scientific construct that is influenced by the values of those who define it [26–30]. Moreover, they put human agency in the face of threats at the centre of their research and acknowledged that this capacity is shaped by access to various capitals, as defined by Bourdieu [29]. They, thus define social resilience as, ‘*the capacity of actors to access capitals in order to - not only cope with and adjust to adverse conditions (i.e. reactive capacity) - but search for and create options (i.e. proactive capacity), and thus develop increased competence (i.e. positive outcomes) in dealing with a threat*. *Access to economic, social and cultural capitals is to a large extent structured by power-related symbolic capital*’ ([25]: p. 289–290).

While the concept of sexual and reproductive health covers a broad range of issues around pregnancy, this study focused on teenage pregnancy among adolescent girls aged 15 to 19 years, in two urban and two rural areas in Ghana (Accra and Begoro) and Tanzania (Dar es Salaam and Mtwara Town). It is widely acknowledged that reproductive health research should target women and men [31], however, this study focused on female adolescents as the individuals most immediately and directly impacted by teenage pregnancy. This study examined whether and how actors (family, peers, teachers, etc.) as well as institutions and organizations (health services, schools, youth development projects, etc.) influence adolescent women’s competence in avoiding or dealing with teenage pregnancy. While the study used quantitative and qualitative methods, this article presents the quantitative results from the urban site in Dar es Salaam, Tanzania. Findings from the urban setting in Ghana have been presented elsewhere [32]. Qualitative studies were also conducted in Tanzania and Ghana to complement the quantitative findings. These results are reported elsewhere [33].

### Conceptual framework

**Fig. 1:**
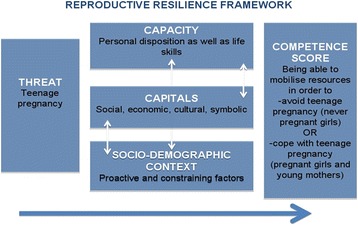
Reproductive resilience framework (modified multi-layered social resilience framework by Obrist, Pfeiffer & Henley, 2010)

This study started from the dominant public health assumption that unwanted and unplanned pregnancy may threaten the health, education, and socio-economic well-being of adolescent women [3, 9]. Thus, the project focused on unwanted and unplanned teenage pregnancy as a potential threat to female adolescents. Drawing on the social resilience approach [25], the study examines how adolescent women build resilience to this threat at the household level, with a particular emphasis on 1) their social, economic, cultural and symbolic capital; 2) their personal capacities; 3) their socio-demographic context; and 4) the outcome: competence (ability to competently deal with the threat of teenage pregnancy) (Fig. 1).

Building on the work of Pierre Bourdieu [29, 30], capital is understood as material and non-material resources that determine human agency. He distinguishes four types of capital. Economic capital refers to the command over economic resources such as cash and assets. Social capital is defined as the various kinds of valued relations with significant others. Cultural capital is divided into three forms: embodied (personal dispositions and habits), objectified (knowledge and tradition stored in material forms) and institutionalized (educational qualification). Bourdieu later added symbolic capital (honor, recognition, and prestige) — a power-related resource that influences the ways in which actors can access other forms of capital. Capital is continuously attained, transferred, transformed, and repositioned. Although this short description simplifies Bourdieu’s work, it allows for a conceptualization of social resilience [25].

According to Bourdieu [29, 30], individual actors are constrained by structures but, at the same time, can re-shape existing structures. The human capacity to reflect and act in relation to a threat is both structured by and structures economic, social, and cultural capital. The definition of capacities moves beyond the common concept of attributes and personal dispositions used in psycho-social resilience scales [34] and allows for the inclusion of life skills. Life skills refer to various abilities for adaptive behavior [35] such as the capacity to decide freely with whom to have sex.

In line with the social resilience approach, the study focused on competence as an outcome of resilience. In order to highlight the re- and pro-active component of social resilience, the competence score was based on the following outcome: *being able* to competently mobilize resources in order to avoid teenage pregnancy (for never pregnant adolescents) or to cope with teenage pregnancy (for pregnant girls and young mothers).

This paper focuses on the role of different types of capital and its impact on the competence score. It is hypothesized that:

1. Adolescents can develop competencies for dealing with the threat of teenage pregnancy.
2. Mobilizing capital (economic, social, cultural, and symbolic resources) increases adolescents’ competence in avoiding or dealing with teenage pregnancy.

## Methods

### Design

The study focused on female adolescents aged 15–19 years, who form part of the older adolescent age group according to the World Health Organization (WHO) [36]. In this article, the terms “adolescents” and “girls” are used interchangeably and refer to the WHO definition of older adolescents. A cross-sectional survey was conducted with female adolescents from Dar es Salaam, Tanzania. In order to gain insights into female adolescents’ resilience, the girls were asked a series of questions on how they dealt with sexuality, unwanted/ unplanned teenage pregnancy, and teen motherhood (Table 1). The questionnaire used pre-coded multiple responses.

**Table 1:** Reproductive resilience research design^a^

Variables	Questions (selection of few examples)
1. Socio-demographic background	
Socio demographic background	How old are you? Are you in a relationship?
*2. Capitals*	
*2.1 Social capital*	*Do you have someone you can turn to in case you have questions related to avoiding/dealing with teenage pregnancy? Whom do you turn to?*
*2.2 Cultural capital*	*Do you have access to other information sources in order to learn about how to avoid/deal with teenage pregnancy? What kind of sources?*
*2.3 Economic capital*	*Do you have someone you can turn to in case you need money to avoid/deal with teenage pregnancy? Whom do you turn to?*
*2.4 Symbolic capital*	*Do you feel accepted within your social environment? Do you strive for a good reputation?*
3. Capacities	
3.1 Psycho-social dispositions	Do you belief that you can successfully manage to avoid/deal with teenage pregnancy? Do you have the ability to establish and maintain relationships to people?
3.2 Life skills	Do you know how to protect yourself from pregnancy? Do you decide freely if, when and with whom you want to have sex?
*4. Competence score*	
*Competence*	*Have you mobilized any social/economic/cultural support in order to actively avoid teenage pregnancy/deal with teenage pregnancy?*

^a^This paper focuses on the impact of capitals (highlighted in italics) on competence (highlighted in italics)

### Study setting and sampling

Dar es Salaam is the largest city in Tanzania. It is divided into three municipalities: Kinondoni, Ilala, and Temeke. According to the 2012 census, Dar es Salaam Region has a population of 4,364,541 ([37]: p. 2). Although Dar es Salaam is not the official capital city, it is the largest city in the country.

**Map 1:**
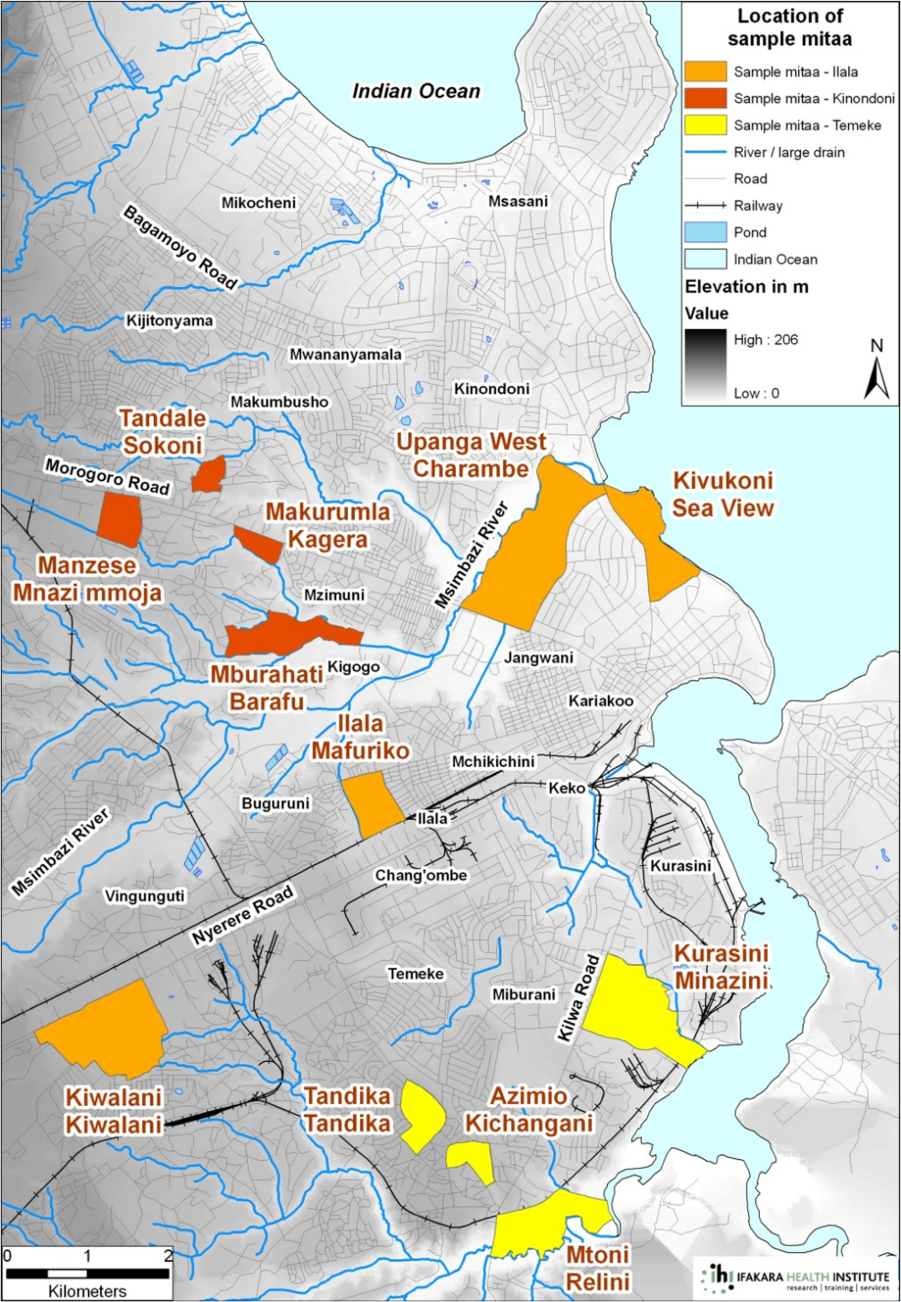
Selected administrative units (Mitaa) in the city of Dar es Salaam

A total of 750 respondents were included in the study. To attain the required sample size for the study, taking into account the female adolescent population (160′266) and teenage pregnancy rate (15%) in Dar es Salaam, 12 clusters — four in each of the three municipalities in Dar es Salaam (Map 1) — were randomly selected. Sample size considerations were based on recommendations that a logistic regression analysis should have at least 10 cases for each explanatory variable [28]. It was estimated that aproximately 30% of respondents would be considered cases (‘high competence’, see Data Analysis section for details) and that approximately 20 variables would be included in the final multivariate regression model. In addition, a 10% non-response rate was allowed.

A one-stage cluster sampling approach was used to select respondents in Dar es Salaam. The second smallest administrative units in the city, the sub-wards or *Mtaa/Mitaa* (Kiswahili for “street/streets”), were used as clusters in all three municipalities (Kindondoni, Ilala and Temeke). Twelve clusters, four in each of the three municipalities in Dar es Salaam (Map 1), were randomly selected. Within each cluster, all households with female adolescents aged 15–19 years were visited and, based on their willingness to participate, they were included in the study [38, 39]. The research team stopped going to households only after all clusters were covered completely. Married girls could be well covered through this approach as most of them either stayed with their family or their partner’s family. In each cluster, an average of 63 adolescents were sampled. At the end of the fieldwork, only six girls could not be included in the study because their caretakers refused to give consent.

### Data generation

Prior to data collection the questionnaire was pre-tested and revised accordingly. From November 2010 to January 2011, data collection was conducted by adolescent women, aged 19 to 24 years. A peer-to-peer data collection approach was chosen to reduce age-related social barriers and to increase trust between interviewees and interviewers. The six data collectors were trained during a three-day training workshop in Dar es Salaam and supervised during data collection. Continous monitoring of data collection and weekly exchanges between project leader, field supervisor, and data collectors aimed to guarantee quality.

### Data analysis

A cumulative competence scale was developed as outcome to assess reproductive resilience. The cumulative score was computed based on 10 competence indicators that refer to the re- and pro-active mobilization of resources. Depending on pregnancy status (never pregnant vs. pregnant adolescents and young mothers), a set of questions related to competence were asked (Table 1). The questions were carefully discussed within the research team, comprising Tanzanian, Ghanaian, and Swiss social scientists, and statisticians as well as Tanzanian adolescent data collectors. Particular attention was given to the local context, including suitability of terms and local concepts of teenage pregnancy. The questions were pre-tested and discussed with Tanzanian adolescents and revised thorougly.

Each competence question answered with ‘yes’ (i.e. having actively mobilized resources, continued education, etc.) contributed ‘1’; questions answered with ‘no’ (i.e. not having actively mobilized resources, did not continue with education, etc.) contributed ‘0’ to the score. Each respondent among the pregnant girls and young mothers could score a minimum of 0 (all questions answered with ‘no’) and a maximum of 10 (100%) (all questions answered with ‘yes’). For the purpose of this analysis, a score of ≤50% was indicative of ‘low competence in mobilizing resources to avoid or deal with pregnancy’, while a score of 51–100% was considered as ‘high competence in mobilizing resouces to avoid or deal with pregnancy’. A 50% cut-off point was used in order to learn about the broad spectrum of competencies among respondents. No reliability testing was performed for score development.

Statistical analyses were conducted using IBM SPSS Statistics 19 and included descriptive statistics, chi-square tests, as well as univariate and multivariate logistic regressions. Bivariate relationship between competence score and capital variables, competence score and ability variables as well as competence score and demographic variables were computed. Variables for the logistic models to identify determinants of resilience were identified by suggestive bivariate relationships. All logistic regression models were controlled for age, since proportionally more never pregnant girls were in the younger age than the pregnant/young mothers. The outcome variable of the study was the ability to avoid pregnancy or cope well with it, which was expressed in the competence scores.

The logistic regressions were fitted to assess the effect of social, cultural, economic, and symbolic capital variables on the odds of being in a high vs. low competence score category. All variables with *p* ≤ 10% in univariate analyses were entered in the mulitviariate model. Analyses were conducted separately by pregnancy status in order to gain insights into the different health realities of non-pregnant female adolescnts compared to pregnant girls and young mothers. Only results from the multivariate logistic regression analysis are presented in this paper.

## Results

### Socio-demographic context of respondents

Of the 750 sampled teenagers, 16% (*n* = 112) reported that they were pregnant or already mothers. Table 2 shows respondents’ sociodemographic characteristics by pregnancy status. Younger girls were more likely to be categorized as never pregnant and not in a relationship. Most of the married girls reported to be pregnant before marriage. Adolescents with less education were more likely than better-educated girls to have started bearing children. At the same time, the data indicates that early childbearing leads to school dropouts. Contrary to Ghana, where a similar study was conducted [32], the education policy in Tanzania until recently stated that girls who became pregnant in school were to be expelled and not allowed to return following their pregnancy. In 2009, the “Law of the Child” Act, 351 was passed by the Tanzanian parliament, which amends this policy and allows girls to return to school. However, it is still unclear whether it will actually be enforced.

**Table 2:** Socio-demographic characteristics of respondents, by pregnancy status

Categories	Never pregnant girls		Pregnant girls and/or young mothers	
	*n* (*N* = 638)	%	*n* (*N* = 112)	%
Age				
15 Years	167	26.2	1	0.9
16 years	107	16.8	11	9.8
17 years	114	17.9	8	7.1
18 years	108	16.9	20	17.9
19 years	142	22.3	72	64.3
Education				
Primary education	277	43.4	85	75.9
Secondary education	326	51.3	21	18.8
Vocational training	13	2.0	0	0
No education	21	3.3	6	5.4
Relationship status				
Single	367	57.5	11	9.8
In a relationship/not married	258	40.4	57	50.9
Married	13	2.0	38	33.9
Divorced/separated	0	0	6	5.4
Both parents of respondent living together with their children				
Yes	372	58.3	53	47.3
No	169	26.5	43	38.4
Others (dead, don’t know)	87	15.2	16	14.3
Respondent’s father has more than one wife				
Yes	213	33.4	37	33.0
No	327	51.3	58	51.8
Others (dead, don’t know)	98	15.4	17	15.2
Religion				
Christians	207	32.4	27	24.1
Muslims	428	67.1	85	75.9
Others	3	0.5	0	0

### Competence of female adolescents

Both groups of adolescent women, especially those who were pregnant or already mothers, had high competence scores. About half of the women who had never been pregnant fell into the category of ‘high competence’ (50.9%), meaning they could get the information and support they needed to avoid pregnancies. Among pregnant women and young mothers, even more respondents had ‘high competence’ levels (70.5%) compared to the female adolescents you had never been pregnant; the majority stated that they know how to avoid or deal with health problems that might affect them or their babies and could get the information and support to do so.

### Social Capital

**Fig. 2:**
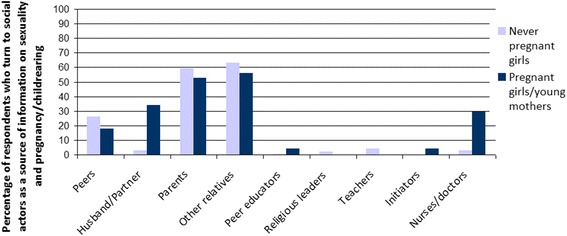
Access to social actors by pregnancy status

Depending on their pregnancy status, female adolescents in Dar es Salaam turned to different social actors, mainly their parents and peers, when in need of information on how to avoid or deal with teenage pregnancy. In former times, other relatives —aunts, in particular — were approached, but due to societal changes and new family structures (moving from extended to nuclear families because of modernity and increasing mobility) parents are increasingly contacted [12, 40, 41]. This transition was confirmed by both groups in the study (Fig. 2). However, Table 3 shows that parents/guardians in Dar es Salaam did not contribute significantly to female adolescents’ competence level. Interviewed girls in Dar es Salaam did not talk to their peers (22% never pregnant girls; 18% pregnant girls and young mothers) about sexual matters as often as to their parents or relatives (51% never pregnant girls; 53% pregnant girls and young mothers). Although it can be concluded that individual actors can contribute to the competence of girls, the quality of information in terms of accuracy and reliability was not covered in this study.

**Table 3:** Multivariate logistic regression analysis: Estimated effect of social, cultural and economic capital on the competence score, by pregnancy status

	*Never pregnant girls (N = 638)*				*Pregnant girls and/or* *young mothers (N = 112)*			
	OR	95%	C.I.	*p*-Value	OR	95%	C.I.	*p*-Value
*Social capital*	1.40	0.875	2.25	0.159				
Peers^b^	1.71	1.18	2.48	**0.004**				a
Partner^b^	2.07	0.76	5.65	0.157				a
Parents/guardians^b^	1.34	0.97	1.86	0.077				a
Other relatives^b^	0.87	0.62	1.22	0.414				a
Religious leaders^b^	3.36	0.69	16.36	0.133				a
Teachers^b^	1.68	0.69	4.08	0.258				a
Nurses^b^	1.43	0.51	4.04	0.495				a
*Cultural capital*	1.80	1.06	3.07	**0.029**	3.33	1.15	9.60	**0.026**
Books^c^	1.63	1.05	2.54	**0.031**	2.63	0.30	23.23	0.383
Brochure^c^	1.41	0.75	2.63	0.286				a
Cell Phones^c^	1.82	0.17	19.22	0.617				a
Magazines^c^	1.96	1.38	2.77	**0.001**	2.75	0.88	8.60	0.083
Music songs^c^	3.22	1.88	5.54	**0.001**	1.67	0.17	16.25	0.660
Radio^c^	1.70	1.18	2.45	**0.004**	3.44	1.35	8.76	**0.010**
Television (TV)	0.91	0.62	1.34	0.633	1.32	0.52	3.35	0.561
*Economic capital*	0.85	0.39	1.88	0.692				
Peers ^d^	3.41	1.73	6.70	**0.001**	1.24	0.22	7.01	0.809
Partner ^d^	2.08	1.18	3.68	**0.012**	3.24	3.24	8.61	**0.019**
Parents/guardians ^d^	1.31	0.90	1.01	0.162	2.98	2.98	7.58	**0.022**
Other relatives ^d^	1.11	0.80	1.54	0.539	3.23	3.23	8.52	**0.018**
Religious leaders ^d^				a				a
Teachers ^d^	10.67	1.35	84.17	**0.025**				a
Nurses ^d^				a				a

^a^The variable was not entered in the logistic regression model as it was not significant according to the variable selection strategy. All variables significant at the 10% level in univariate analyses were considered candidates for the mulitviariate model

^b^Spontaneous mention of social actors never pregnant girls and pregnant girls/young mothers turn to for information on sexuality and teenage pregnancy/childrearing

^c^Spontaneous mention of mass media never pregnant girls and pregnant girls/young mothers turn to for information on sexuality and teenage pregnancy/childrearing

^d^Spontaneous mention of social actors never pregnant girls and pregnant girls/young mothers turn to for financial support related to avoiding teenage pregnancy or dealing with teenage pregnancy/childrearing

### Cultural Capital

In a context where family members, partners, and peers may not offer reliable information about sexual health and pregnancy, additional sources of information such as mass media become important for youth [9]. A logistic regression including social (having someone to turn to for social support), economic (having someone to turn to for economic support), cultural (having access to other sources of information), and symbolic capital (being accepted by others) showed that only cultural capital contributed to the competence of never pregnant girls [OR = 1.80, 95% CI = 1.06 to 3.07, *p* = 0.029] as well as to pregnant adolescents and young mothers [OR = 3.33, 95% CI = 1.15 to 9.60, *p* = 0.026] (Table 3). This finding highlights the importance of cultural capital as a determinant for reproductive resilience. Table 3 provides information about the contribution of different types of media to the competence of young people. Music and the Tanzanian magazines, *Fema* and *Si Mchezo!,* published by Femina HIP, contributed significantly to the competence of never pregnant girls. Femina HIP is the biggest local multimedia platform and civil society organization working with youth, communities, and partners in Tanzania. It publishes *Fema* and *Si Mchezo!*, the top two magazines in Tanzania for 15 to 25 year olds. Catering to secondary school students and written in English and Swahili, *Fema* is published quarterly and has a circulation of 180,000. Directed to out-of-school youth and written in Swahili, *Si Mchezo!* is published on a bi-monthly basis and has a circulation of approximately 175,000. The two magazines also seek to reach young people in the workplace and in organizational settings.

**Fig. 3:**
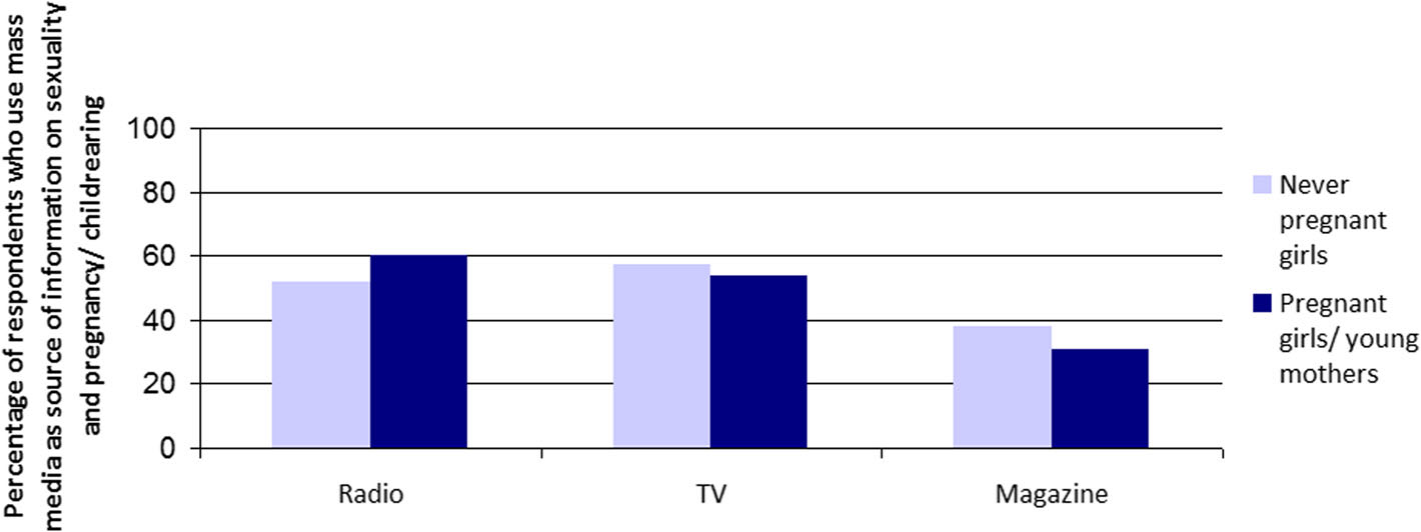
Access to mass media by pregnancy status

Radio contributed to the competence of both groups, but not everyone has access to cultural capital. The findings show that 57% of never pregnant girls and 54% of their pregnant peers and young mothers had access to television (TV) (Fig. 3). Radio was used widely among young mothers or mothers to be, with 60% saying that they listen to it, while 52% of the never pregnant respondents mentioned it. Both youth magazines published by Femina HIP were widely read; 38% of the never pregnant and 31% of the pregnant girls/young mothers stated that they read one of these magazines (Fig. 3).

While different types of media contributed to the competence of never pregnant girls, the same could not be observed for pregnant girls and young mothers, as mass media campaigns tend to target girls who are not pregnant.

### Economic Capital

Access to economic resources in general did not significantly contribute to the competence of female adolescents [never pregnant girls: OR = 0.85, 95% CI = 0.39 to 1.88, *p* = 0.692; for pregnant girls and young mothers, the variable “economic capital” could not be entered in the regression model as it was not significant in univariate analysis] (Table 3). However, the analysis of the role of individual actors illustrated that pregnancy status considerably changed the way social actors matter for adolescent girls (Table 3). For never pregnant girls, economic capital mobilized through social actors outside the family contributed to their competence, while for pregnant girls and young mothers, economic support from family members contributed to their competence.

### Symbolic Capital

Results show that almost all interviewed respondents aimed to establish symbolic capital. Some 99% (*n* = 634) of the never pregnant girls and 98% (*n* = 110) of the pregnant girls and young mothers ‘strived for a good reputation’. In addition, regardless of their pregnancy status almost all female study participants (99%, *n* = 634 of the never pregnant girls; 98%, *n* = 110 of the pregnant girls/young mothers) reported feeling accepted in their community. This finding highlights the importance of maintaining symbolic capital. Surprisingly, the symbolic capital of both groups of female adolescents did not significantly contribute to the competence score. Although this presentation simplifies Bourdieu’s conceptualization of symbolic capital, it helps to provide insights into the different dimensions of social resilience. While findings indicate that symbolic capital does not contribute to the competence of female adolescents, it is also acknowledged that it is not easy to quantitatively capture symbolic capital in all its complexity.

## Discussion

While acknowledging that teenage pregnancy often has serious negative consequences for female adolescents, this study highlights that common portrayals of adolescent mothers as unprepared ‘children, who have children’ might not hold true for all girls [1–3, 42, 43]. Our study shows that, in contrast to public health perceptions of teenage motherhood and in spite of the challenges some youth face, adolescent girls are not mere victims per se but also social actors that try to mobilize resources actively to secure their own health and that of their child. The higher competence score of pregnant girls and young mothers compared to peers who had never been pregnant might reflect a dynamic learning process. Due to pregnancy and motherhood, female adolescents must develop support networks and knowledge of resources in response to their situation. Thus, childbirth might be looked at as a ‘*turning point experience*’ [17: p. 136] that offers new opportunities to break away from the past [43]. What might be regarded as a threat in one situation becomes a protective factor in another. This depends on how threats and protective factors are perceived by the affected actors [43]. The psycho-social resilience literature highlights that major life transitions provide new opportunities for resilience, not only for young mothers but also for young fathers [44–47]. Findings point to factors that can further support adolescents in dealing with teenage pregnancy.

Findings show that parents/guardians in Dar es Salaam did not contribute significantly to female adolescents’ competence level. A literature review [40] of parent-child communication about sexuality in sub-Saharan Africa argues that discussions on sexual matters tend to be authoritarian and vague as parents are often overwhelmed with their new roles and do not know how to provide sex education. Talking to peers significantly contributed to the competence of girls who had not been pregnant. Peers are often regarded as crucial in terms of shaping adolescent norms that serve as reference points for decision making related to health [41]. Peer communication is characterized by an open atmosphere and less restricted by cultural norms and taboos.

Cultural capital, defined as the internalization of cultural values around teenage pregnancy that are represented in media such as TV, radio or magazines, can contribute to competence. Nichter [14] points out that local people respond practically to new information and resources, and they do so within the framework of their cultural institutions or knowledge. Using mass media is one way to make sure that adolescent women get accurate information and make informed decisions.

This study could not confirm access to economic capital as the most important driving factor [48–50]. Cultural and social factors, especially for never pregnant girls, are equally important. This finding promotes the value of the social development debate that highlights the role of social and cultural institutions and organisations for development, in general, and for building sexual and reproductive health knowledge, in particular. Youth-focused magazines or TV programs, for instance, can build competence. However, one needs to carefully analyze who benefits from such interventions and who falls through the cracks.

### Reflection on the reproductive resilience framework

The application of the reproductive resilience framework, which builds on the social resilience approach [25], proved to be useful to this study in several respects. First, the framework provides a clear definition of and highlights socio-economic and cultural aspects of reproductive resilience, thereby allowing us to move beyond existing ecological or child-development resilience concepts. Focusing on the strengths of people rather than on their weaknesses alone opens new possibilities for dealing with and reducing threats. The framework acknowledges the learning, self-organization, and creative potential of people and institutions that might be able to actively deal with threats.

Second, the framework highlights that resilience is not just about psychological traits that individuals are born with but rather that people can develop competencies that allow them to deal with a given threat. The data presented here illustrate how crucial it is to understand how and by whom these capacities are built in order to identify entry points for future interventions. This study does not, however, provide insights into whether or how these capacities are then translated into action.

Third, the framework provides new insights beyond the social or economic environment. This study makes it clear that cultural capital in terms of knowledge passed on by social and cultural institutions and organizations can significantly contribute to resilience and therefore needs to be considered in detail in resilience research.

While the reproductive resilience framework presented here offers new insights, it also has some shortcomings. The framework is grounded in the applied realm and therefore runs the risk of reducing resilience to normative and dominant concepts at the expense of the perspectives of actors [22, 25]. Although this study focused on unwanted/unplanned pregnancy, some involved female adolescents might have looked at teenage pregnancy as something that could improve their symbolic capital (their status/prestige in their community). There is clearly a need to complement experts’ views of the key categories (threat, capitals, capacities and competencies) with qualitative research into local meanings corresponding with these scientific constructs.

To cover pro-active capacities, never pregnant girls were included in the study. As in other studies, these girls were interviewed in the absence of a threat that had occurred already [51, 52], which might lead to overlooking the actual context of coping with adversity ([53]: p. 754).

The study presented here used a cumulative competence scale to provide a snapshot of teenage pregnancy-related realities of girls in the urban centre of Dar es Salaam. As resilience is not a stable and durable phenomenon, longitudinal studies would be best suited to collecting reliable information [53: p. 755]. Survey data provide verifiable and generalizable data [54] but they can only generate a fraction of the information needed to understand the complexity of social institutions and relations in which reproductive practices are contextualized [55]. The quality of information adolescents received while mobilizing different capitals, in terms of accuracy and reliability, was, for instance, not covered in this study and would require deeper quantitative as well as qualitative investigation. In addition, factors other than resilience might account for the differences in the “competence scores” of female adolescents presented here. Quantitative approaches alone are not sufficient to provide insight into the full potential of agency and related creativity of actors in different contexts. Adolescent competencies in urban areas can only be adequately comprehended by understanding their everyday life, highlighting the need for culturally-adapted, mixed-methods studies.

## Conclusions

This article examined how various material and non-material resources can contribute to the competence of adolescents to avoid teenage pregnancy or to successfully deal with it. The reproductive resilience framework provided new insights into the resources and competencies of girls in Dar es Salaam, Tanzania. Although teenage pregnancy and motherhood involves various challenges and risks, many interviewed female adolescents tried to deal with it actively and might turn some of the challenges into opportunities [33, 56–60]. Strengthening the factors, that contribute to building competence in female adolescents, such as providing information in an appealing way through magazines, are important entry points for future interventions.

## Abbreviations

TV: Television
UN: United Nations
WHO: World Health Organization
